# The Effect of Frailty versus Initial Glasgow Coma Score in Predicting Outcomes Following Chronic Subdural Hemorrhage: A Preliminary Analysis

**DOI:** 10.7759/cureus.10048

**Published:** 2020-08-26

**Authors:** Matthew K McIntyre, Cameron Rawanduzy, Adil Afridi, Jesse A Honig, Mohamed Halabi, Jake Hehir, Meic Schmidt, Chad Cole, Ivan Miller, Chirag Gandhi, Fawaz Al-Mufti, Christian A Bowers

**Affiliations:** 1 Department of Neurological Surgery, Oregon Health & Science University, Portland, USA; 2 Department of Neurosurgery, New York Medical College, Valhalla, USA; 3 Department of Neurosurgery, University of New Mexico, Albuquerque, USA; 4 Department of Neurosurgery, Westchester Medical Center, Valhalla, USA; 5 Department of Emergency Medicine, Westchester Medical Center, Valhalla, USA

**Keywords:** modified frailty index, subdural hemorrhage, charlson comorbidity index, mortality, age, gcs

## Abstract

Background

Initial Glasgow Coma Score (iGCS) is a well-known predictor of adverse outcomes following chronic subdural hemorrhage (cSDH). Frailty, i.e. a reduced physiologic reserve, is associated with poorer outcomes across the surgical literature, however, there is no consensus on the best measure of frailty. To date, no study has compared frailty’s ability to predict cSDH outcomes versus iGCS. The goal of this study was to, therefore, examine the prognostic value of the 5- (mFI-5) and 11-factor (mFI-11) modified frailty index, and Charlson Comorbidity Index (CCI) versus iGCS following cSDH.

Methods

Between January, 2016 and June, 2018, patients who presented to the emergency department with cSDH were retrospectively identified using the International Classification of Diseases (ICD) codes. mFI-5, mFI-11, and CCI scores were calculated using patient baseline characteristics. Primary endpoints were death and discharge home and subgroup analyses were performed among operative cSDH. Univariate and multivariate logistic regressions were used to determine predictors of primary endpoints.

Results

Of the 109 patients identified, the average age was 72.6±1.6 years and the majority (69/109, 63.3%) were male. The average CCI, mFI-5, and mFI-11 were 4.5 ±0.2, 1.5 ±0.1, and 2.2 ±0.1, respectively. Fifty (45.9%) patients required surgical intervention, 11 (10.1%) died, and 48 (43.4%) were discharged home. In the overall cohort, while the only multivariate predictor of mortality was iGCS (OR=0.58; 95%CI:0.44-0.77; p=0.0001), the CCI (OR=0.73; 95%CI:0.58-0.92; p=0.0082) was a superior predictor of discharge home compared to iGCS (OR=1.46; 95%CI:1.13-1.90; p=0.0041). Conversely, among those who received an operative intervention, the CCI, but not iGCS, independently predicted both mortality (OR=4.24; 95%CI:1.01-17.86; p=0.0491) and discharge home (OR=0.55; 95%CI:0.33-0.90; p=0.0170). Neither mFI nor age predicted primary outcomes in multivariate analysis.

Conclusion

While frailty is associated with worse surgical outcomes, the clinical utility of the mFI-5, mFI-11, and CCI in cSDH is unclear. We show that the iGCS is an overall superior predictor of mortality following cSDH but is outperformed by the CCI after operative intervention. Similarly, the CCI is the superior predictor of discharge home in cSDH patients overall and following an operative intervention. These results indicate that while the iGCS best predicts mortality overall, the CCI may be considered when prognosticating post-operative course and hospital disposition.

## Introduction

Chronic subdural hemorrhage (cSDH) is an increasingly common pathology encountered in modern neurosurgical practice given its high frequency among the elderly [1]. The outcomes following cSDH are generally positive, but one large, recent cohort study found that age is correlated with poorer functional outcomes and lower rates of discharge home, and the majority of patients with cSDH are in their 80s [2]. While both advanced chronological age and low initial Glasgow Coma Score (iGCS) are established predictors of poorer outcomes following cSDH, [2] there is a paucity of information regarding the effect of a patients’ underlying comorbidities on cSDH prognosis.

Frailty, i.e. a reduced physiologic reserve, is an emerging concept across the surgical literature that aims to elucidate the differential effects of age versus the cumulative effect of multiple comorbidities on surgical outcomes. While frailty, as most often measured by the modified frailty index (mFI-11), has been associated with poorer neurosurgical outcomes, its effect among those with cSDH is unclear [3-6]. To date, no study has compared the effect of frailty versus iGCS for predicting outcomes in both operative and non-operative cSDH. Complicating matters further, with over 215 different frailty indices in the literature [7], there remains no consensus regarding which frailty index best predicts outcomes; an effect that may be pathology and patient-population specific. Other common measures of frailty are the Charlson Comorbidity Index (CCI) and the new 5-factor modified frailty index (mFI-5). As such, the goal of this study is to perform a comparative analysis of multiple measures of frailty including the CCI, mFI-5, and mFI-11, versus iGCS for predicting mortality and discharge location among both operative and non-operative cSDH patients.

## Materials and methods

Study design and setting

This retrospective study was performed between January 2016 and June 2018 at a quaternary academic referral center (Westchester Medical Center) with high neurosurgical volume. Institutional Review Board Approval with a waiver of informed consent was obtained from New York Medical College & Westchester Medical Center (IRB 12921). All data were retrospectively collected using the electronic medical record system by trained and monitored data abstractors.

Subject selection

cSDH patients were identified by reviewing the International Classification of Diseases (ICD) codes for subdural hemorrhage for all patients presenting to our emergency department in the study period. Inclusion criteria were patients 14 years or older who presented with a chronic subdural hemorrhage. Patients were excluded if they had a history of (non-subdural related) neurosurgical procedure or were found to not have a chronic SDH on in-house imaging. We defined cSDH as any subdural hemorrhage with a chronic component including mixed, acute on chronic, pure chronic, or subacute SDH.

Measures and endpoints

For each patient, demographics, smoking and alcohol abuse history, anti-coagulant or anti-platelet (AC/AP) drug use, GCS score, chief complaint, and cSDH characteristics (thickness, prior SDH), and presence of an isolated head injury were collected. The eleven-factor modified frailty index (mFI-11) was calculated by assigning one point for the presence of each of the following pre-hemorrhage characteristics for a maximum of 11 points: hypertension requiring medication, congestive heart failure, myocardial infarction, previous percutaneous coronary intervention or angina, transient ischemic attack or cerebrovascular accident without neurological deficit, cerebrovascular accident with neurological deficit, peripheral vascular disease or ischemic chest pain, chronic obstructive pulmonary disease or current pneumonia, diabetes mellitus, non-independent functional status, and impaired sensorium (Table 1). Non-independent functional status was defined as requiring assistance from another person for activities of daily living [8-11]. The five-factor modified frailty index (mFI-5) was defined similarly but with the presence of hypertension requiring medication, congestive heart failure, chronic obstructive pulmonary disease or current pneumonia, diabetes mellitus, or non-independent functional status [12,13]. The CCI was calculated as described extensively in prior literature (Table 2) [14,15].

**Table 1:** 11- and 5-factor modified frailty index (mFI) characteristics and prevalence in patients with chronic subdural hemorrhage (cSDH)

History of:	mFI-5	mFI-11
Hypertension on medications	77 (70.6%)	
Congestive Heart Failure	14 (12.8%)	
Diabetes mellitus	24 (22.0%)	
Chronic Obstructive Pulmonary Disease, Pneumonia	9 (8.3%)	
Non-independent functional status	35 (32.1%)	
History of transient ischemic attack or cerebrovascular accident without neurological deficit	-	17 (15.6%)
Myocardial Infarction	-	9 (8.3%)
Peripheral Vascular Disease or ischemic rest pain	-	5 (4.6%)
Cerebral vascular accident with deficit	-	9 (8.3%)
Previous coronary intervention or angina	-	24 (22.0%)
Impaired Sensorium	-	21 (19.3%)

**Table 2:** Charlson Comorbidity Index (CCI) characteristics and prevalence in patients with chronic subdural hemorrhage (cSDH)

Characteristic			Points	Prevalence
Age (years)				
	<50		0	6 (5.5%)
	50-59		1	18 (16.5%)
	60-69		2	20 (18.3%)
	70-79		3	17 (15.6%)
	$\geq$80		4	48 (44.0%)
Myocardial Infarction			1	9 (8.3%)
Congestive Heart Failure			1	14 (12.8%)
Peripheral Vascular Disease			1	5 (4.6%)
Cerebrovascular Accident or Transient Ischemic Attack			1	17 (15.6%)
Dementia			1	19 (17.4%)
Chronic Obstructive Pulmonary Disease			1	9 (8.3%)
Connective Tissue Disease			1	3 (2.8%)
Peptic Ulcer Disease			1	5 (4.6%)
Liver Disease				
	Mild		1	3 (2.8%)
	Moderate to Severe		3	5 (4.6%)
Diabetes Mellitus				
	Uncomplicated		1	22 (20.2%)
	End organ damage		2	2 (1.8%)
Hemiplegia			2	2 (1.8%)
Moderate to Severe Chronic Kidney Disease			2	14 (12.8%)
Solid tumor				
		Localized	2	13 (11.9%)
		Metastatic	6	1 (0.9%)
Leukemia			2	3 (2.8%)
Lymphoma			2	1 (0.9%)
AIDS			6	0 (0.0%)

Primary endpoints were discharge home and death. Secondary endpoints needed for surgical intervention (craniotomy, burr hole craniotomy, or subdural evacuating port system (SEPS) treatment), hospital length of stay (hLOS), ICU length of stay (ICU-LOS), deep vein thrombosis (DVT), pulmonary embolism (PE), pneumonia, tracheostomy, gastrostomy, and discharge GCS (excluding deaths).

Statistical analysis

Normal distributions were determined using an Anderson-Darling normality test. T-tests were used for normally distributed continuous samples and Mann-Whitney tests were used for non-normally distributed continuous samples. Continuous data were shown using mean ± standard error of the mean (SEM). Fisher’s exact tests were used for binary variables and odds ratios (OR) are shown with 95% confidence intervals (95% CI). Subgroup analyses were conducted among those who required an operation. Similar to prior studies, multivariate logistic regressions were performed via the enter method using known predictors of outcomes sex, prior subdural hemorrhage, craniotomy, AC/AP use, iGCS, and SDH thickness followed by forward conditional addition of either mFI-11, mFI-5, or CCI to the model. Age was not included due to collinearity with the CCI. No collinearity was detected in either model, as defined as a variance inflation factor of less than one or greater than 10. Statistical analysis was performed using Prism 8.3.0 (GraphPad Software Inc., La Jolla, CA) and SPSS version 25 (IBM Corp., Armonk, NY). Significance was defined as p<0.05.

## Results

Demographics and baseline characteristics

**Figure 1:**
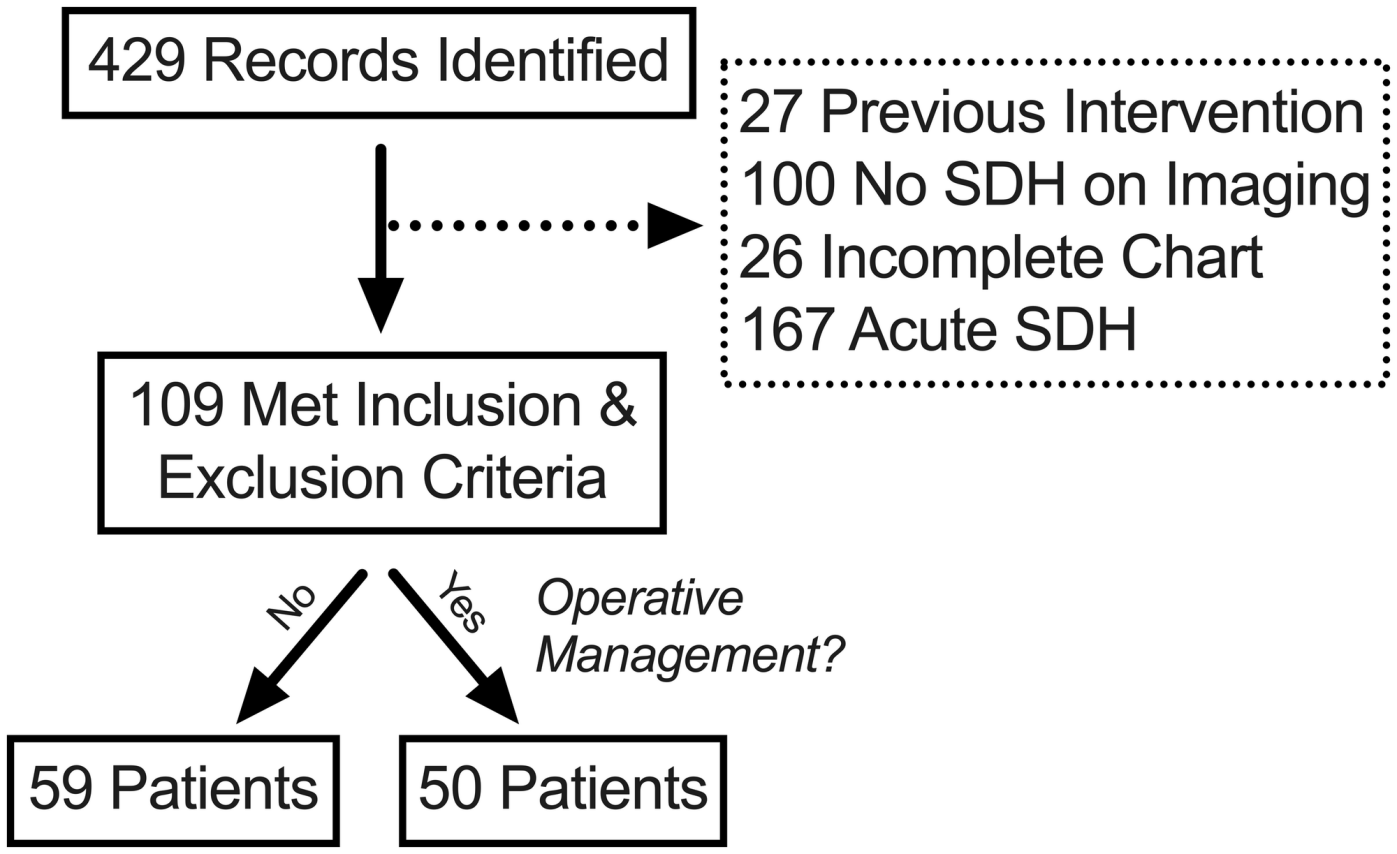
Patient identification and selection SDH: subdural hemorrhage

Of the 429 records identified, 109 met inclusion and exclusion criteria (Figure 1). The average age (range: 14-98 years) of the cohort was 72.6 ±1.6 years, the majority of patients were men (69/109, 63.3%), and 25 (63.3%) had a history of prior subdural hemorrhage (Table 3). As expected, the most common chief complaint was fall (74/109, 67.9%) and half of the patients had taken AC/AP medication prior to hemorrhage (55/109, 50.5%). The average iGCS was 13.4 ±0.3 (range: 3T-15) and the majority of patients had an isolated head injury (100/109, 91.7%). Most hemorrhages were of mixed density (93/109, 85.3%) and the average thickness was 16.0 ±0.7mm. For other baseline characteristics, see Table 3.

**Table 3:** Baseline characteristics and comparison between operative and non-operative patients ^indicates non-normally distributed sample. AC/AP: Anticoagulation/Antiplatelet medication, MVC: Motor Vehicle Collision, iGCS: initial Glasgow Coma Score, cSDH: chronic subdural hemorrhage, CCI: Charlson Comorbidity Index, mFI: modified frailty index.

		Overall (n=109)	Non-operative (n=59)	Operative (n=50)	P-value
Age (years)^		72.6 ±1.6	76.5 ±2.1	67.9 ±2.2	0.0020
Men		69 (63.3%)	39 (57.4%)	30 (60.0%)	0.8509
Prior subdural hemorrhage		25 (22.9%)	14 (23.7%)	11 (22.0%)	>0.9999
Smoking history		39 (35.8%)	24 (40.7%)	15 (30.0%)	0.3167
Alcohol abuse		18 (16.5%)	9 (15.3%)	9 (18.0%)	0.7978
AC/AP		55 (50.5%)	27 (45.8%)	28 (56.0%)	0.3384
Chief Complaint					
	Fall	74 (67.9%)	43 (72.9%)	31 (62.0%)	0.3034
	Altered Mental Status	12 (11.0%)	7 (11.9%)	5 (10.0%)	>0.9999
	MVC	3 (2.8%)	0 (0.0%)	3 (6.0%)	0.0934
	Assault	2 (1.8%)	1 (1.7%)	1 (2.0%)	>0.9999
	Other/unknown	20 (18.3%)	10 (17.0%)	10 (20.0%)	0.8050
iGCS^		13.4 ±0.3	12.9 ±0.5	13.9 ±0.3	0.0423
Isolated head Injury		100 (91.7%)	51 (86.4%)	49 (98.0%)	0.0370
cSDH thickness (mm)^		16.0 ±0.7	13.6 ±0.9	18.8 ±1.0	0.0003
Type of cSDH					
	Mixed Density	93 (85.3%)	49 (83.1%)	44 (88.0%)	0.5900
	Subacute	5 (4.6%)	4 (6.8%)	1 (2.0%)	0.3721
	Pure Chronic	11 (10.1%)	6 (10.2%)	5 (10.0%)	>0.9999
CCI		4.5 ±0.2	5.1 ±0.3	3.8 ±0.3	0.0033
mFI-5^		1.5 ±0.1	1.5 ±0.1	1.4 ±0.1	0.2580
mFI-11^		2.2 ±0.1	2.5 ±0.2	1.9 ±0.2	0.0471

The average CCI was 4.5 ±0.2 (range: 0-11) and 42 (38.5%) had a CCI³6. The most commonly identified CCI characteristic, beyond age, was a history of uncomplicated diabetes (22/109, 20.2%) followed by a history of dementia (19/109, 17.4%) (Table 2). The average mFI-11 (range: 0-7) and mFI-5 (range: 0-4) was 2.2 ±0.1 and 1.5 ±0.1, respectively, with 73 (67.0%) having a mFI-11 ³2 and 55 (50.5%) having a mFI-5 ³2. The most commonly identified characteristic in both the mFI-5 and mFI-11 systems was hypertension on medications (77/109, 70.6%) followed by non-independent functional status (35/109, 32.1%) (Table 1).

Clinical course and outcomes

One hundred and five (96.3%) patients were admitted and 50 (45.9%) required a surgical evacuation of SDH. Five (4.6%) patients developed a DVT, 1 (0.9%) a PE, and 5 (4.6%) pneumonia. Six (5.5%) patients required a tracheostomy tube and 9 (8.3%) a gastrostomy tube. The average ICU-LOS was 5.0 ±0.5 days (range: 0-22) while the hLOS was 9.0 ±0.9 days (range: 1-62). Ultimately, the average discharge GCS was 14.3 ±0.2, a plurality of patients were discharged home (48/109, 44.0%), and 11 (10.1%) patients died (Table 4).

**Table 4:** Patient Outcomes and comparison between operative and non-operative patients ^indicates non-normally distributed sample. hLOS: hospital length of stay, ICU-LOS: Intensive care unit length of stay.

		Overall (n=109)	Non-operative (n=59)	Operative (n=50)	P-value
hLOS (days)^		9.0 ±0.9	8.3 ±1.4	9.7 ±0.9	0.0139
ICU LOS (days)		5.0 ±0.5	4.2 ±0.9	5.7 ±0.7	0.0011
Deep Vein Thrombosis		5 (4.6%)	2 (3.4%)	3 (6.0%)	0.6591
Pulmonary Embolism		1 (0.9%)	0 (0.0%)	1 (2.0%)	0.4587
Pneumonia		5 (4.6%)	2 (3.4%)	3 (6.0%)	0.6591
Tracheostomy		6 (5.5%)	3 (5.1%)	3 (6.0%)	>0.9999
Gastrostomy		9 (8.3%)	5 (8.5%)	4 (8.0%)	>0.9999
Discharge GCS (without death) ^		14.3 ±0.2	14.2 ±0.2	14.5 ±0.2	0.0793
Discharge location					
	Home	48 (44.0%)	26 (44.1%)	22 (44.0%)	>0.9999
	Acute Rehab	30 (27.5%)	9 (15.3%)	21 (42.0%)	0.0025
	Subacute Rehab	6 (5.5%)	4 (6.8%)	2 (4.0%)	0.6853
	Nursing Home	13 (11.9%)	12 (20.3%)	1 (2.0%)	0.0029
Death		11 (10.1%)	7 (11.9%)	4 (8.0%)	0.5439

Clinical course and outcomes: the effect of operative management

Patients who received operative evacuation of SDH were significantly younger (67.9 ±2.2 versus 76.5 ±2.1; p=0.0020), had a higher iGCS (13.9 ±0.3 versus 12.9 ±0.5; p=0.0423), larger SDH thickness (18.8 ±1.0 versus 13.6 ±0.9; p=0.0003), and were more likely to have an isolated head injury (OR=7.7; 95%CI: 1.1-86.9; p=0.0370), indicating careful selection of surgical candidates. Likewise, those that received an operation had decreased CCI (3.8 ±0.3 versus 5.1 ±0.3; p=0.0033) and mFI-11 (1.9 ±0.2 versus 2.5 ±0.2; p=0.0370) but not mFI-5 (p=0.2580) scores compared to those who did not have an operation. There were no significant baseline differences in history of SDH, smoking or alcohol abuse history, AC/AP use, or type of SDH between those who did or did not receive an intervention (Table 3).

As expected, those that received an operation had longer hospital (9.7 ±0.9 versus 8.3 ±1.4 days; p=0.0139) and ICU (5.7±0.7 versus 4.2±0.9days; p=0.0011) length of stay but no differences in complications (p>0.05) (Table 4). Interestingly, there were no differences in rates of discharge home or death between those who did or did not receive surgical drainage. However, patients in the operative group were more likely to be discharged to acute rehabilitation (OR=4.0; 95%CI: 1.7-9.2; p=0.0025), less likely to be discharged to a nursing home (OR=0.08; 95%CI: 0.007-0.48; p=0.0029), and trended to have a higher discharge GCS scores (14.5 ±0.2 versus 14.2±0.2; p=0.0793). Of the patients who received operative evacuation of SDH, 33 (66%) had a craniotomy while 17 (34%) had either a burr hole craniotomy or a subdural evacuation port system device placement. There were no significant differences in any primary or secondary endpoints between the two strategies of operative management (Table 4).

Clinical course and outcomes: the effect of patient frailty

As expected, patients in the CCI$\geq$6, mFI-11$\geq$2, and mFI-5$\geq$2 groups were each older than their non-frail counterparts (Table 5). Those in the CCI$\geq$6 (p=0.0080) and mFI-11 $\geq$2 (p=0.0093) groups also presented with significantly lower iGCS scores. Those in the mFI-5 $\geq$2 group also trended to have decreased iGCS scores compared to those in the mFI-5 $\leq$1 group. As noted above, those that received an operation had significantly lower CCI and mFI-11 scores compared to those that did not receive an intervention (Table 4). Despite this, there were no significant differences in SDH thickness, hospital or ICU LOS, complications, tracheostomy, or gastrostomy tube need. Patients in the mFI-11 $\geq$2 (p=0.0108) and mFI-5 $\geq$2 (0.0186) groups, but not CCI$\geq$6, were discharged with significantly lower GCS scores. There were also significant reductions in rates of discharge home and corresponding increases in discharge to nursing home or hospice in each frailty group. Finally, while there were no significant differences in mortality, there were more deaths in each of the frail groups.

**Table 5:** The effect of frailty on primary and secondary outcomes CCI: Charlson Comorbidity Index, mFI: modified frailty index, iGCS: initial Glasgow Coma Score, hLOS: hospital length of stay, ICU-LOS: Intensive Care Unit length of stay.

		CCI			mFI-11			mFI-5		
		CCI $\leq$5 (n=67)	CCI $\geq$6 (n=42)	P-value	mFI $\leq$1 (n=36)	mFI $\geq$2 (n=73)	P-value	mFI-5 $\leq$1 (n=54)	mFI-5 $\geq$2 (n=55)	p-value
Age (years) ^		66.8 ±2.1	81.8 ±1.6	<0.0001	66.1 ±3.3	75.7 ±1.6	0.0035	69.1 ±2.4	75.9± 1.9	0.0349
iGCS^		13.6 ±0.4	13.0 ±0.4	0.0080	14.2 ±0.4	13.0 ±0.4	0.0093	13.7 ±0.4	13.1 ±0.4	0.0850
SDH thickness		15.6 ±0.9	16.5 ±1.2	0.5550	16.4 ±1.3	15.8 ±0.9	0.6953	16.4 ±1.0	15.5 ±1.0	0.5363
hLOS (days)^		9.7 ±1.2	7.8 ±1.0	0.2720	8.8 ±1.3	9.0 ±1.1	0.7781	7.9 ±1.0	9.9 ±1.4	0.4039
ICU LOS (days)		5.0 ±0.7	4.9 ±0.9	0.3948	4.8 ±0.8	5.0 ±0.7	0.4745	4.7 ±0.7	5.2 ±0.9	0.6792
Deep Vein Thrombosis		4 (6.0%)	1 (2.4%)	0.6470	1 (2.8%)	4 (5.5%)	>0.9999	1 (1.9%)	4 (7.3%)	0.3634
Pulmonary Embolism		1 (1.5%)	0 (0.0%)	>0.9999	0 (0.0%)	1 (1.4%)	>0.9999	0 (0.0%)	1 (1.8%)	>0.9999
Pneumonia		4 (6.0%)	1 (2.4%)	0.6470	1 (2.8%)	4 (5.5%)	>0.9999	1 (1.9%)	4 (7.3%)	0.3634
Tracheostomy		5 (7.5%)	1 (2.4%)	0.4025	1 (2.8%)	5 (6.8%)	0.6616	2 (3.7%)	4 (7.3%)	0.6787
Gastrostomy		6 (9.0%)	3 (7.1%)	>0.9999	3 (8.3%)	6 (8.2%)	>0.9999	6 (11.1%)	3 (5.5%)	0.3203
Discharge GCS (without death)^		14.3 ±0.2	14.5 ±0.2	0.5264	14.7 ±0.2	14.1 ±0.2	0.0108	14.7 ±0.1	14.0 ±0.3	0.0186
Discharge Location	Home	35 (51.5%)	13 (31.0%)	0.0477	22 (61.1%)	26 (35.6%)	0.0144	31 (57.4%)	17 (30.9%)	0.0070
	Acute Rehab	22 (32.8%)	8 (19.1%)	0.1295	9 (25.0%)	21 (28.8%)	0.8204	13 (24.1%)	17 (30.9%)	0.5211
	Subacute Rehab	2 (3.0%)	4 (9.5%)	0.2022	1 (2.8%)	5 (7.9%)	0.4121	3 (5.6%)	3 (5.5%)	>0.9999
	Nursing Home/ hospice	3 (4.5%)	10 (23.8%)	0.0045	1 (2.8%)	12 (16.4%)	0.0566	2 (3.7%)	11 (20.0%)	0.0153
Death		5 (7.4%)	6 (14.3%)	0.3281	2 (5.6%)	9 (12.3%)	0.3325	4 (7.4%)	7 (12.7%)	0.5269

Multivariate logistic regressions

To examine the influence of each frailty measure as predictors of mortality and discharge home, stepwise multivariate logistic regressions were performed while including variables known to be associated with poorer outcomes following cSDH. Age was not included for this analysis due to co-linearity with frailty measures. In multivariate analysis, the single most significant predictor of mortality was the iGCS (OR=0.58; 95%CI: 0.44-0.77; p<0.0001) and was not independently predicted by any frailty measure (Table 6). Discharge home, however, was independently predicted by both iGCS (OR=1.46; 95%CI: 1.13-1.90; p=0.0041) and CCI (OR=0.73; 95%CI: 0.58-0.92; p=0.0082) score but not either mFI.

**Table 6:** Multivariate logistic regressions for primary endpoints among all patients in the cohort CCI: Charlson Comorbidity Index, mFI: modified frailty index, iGCS: initial Glasgow Coma Score, AC/AP: anticoagulation/antiplatelet medication.

	Characteristic	Multivariate OR (95% CI)	P-value
Mortality	Sex	2.03 (0.26-15.60)	0.4961
	Prior SDH	0 (0-infinity)	0.9980
	Craniotomy for SDH	1.46 (0.17-12.62)	0.7310
	AC/AP	1.31 (0.19-8.89)	0.7815
	iGCS	0.58 (0.44-0.77)	0.0001
	SDH Thickness	1.07 (0.92-1.24)	0.3605
	mFI-11	Not Included	0.6560
	mFI-5	Not Included	0.6387
	CCI	Not Included	0.3296
Discharge Home	Sex	0.65 (0.26-1.67)	0.3716
	Prior SDH	0.50 (0.16-1.52)	0.2218
	Craniotomy for SDH	0.46 (0.15-1.38)	0.1647
	AC/AP	1.55 (0.58-4.15)	0.3808
	iGCS	1.46 (1.13-1.90)	0.0041
	SDH Thickness	1.00 (0.93-1.06)	0.8932
	mFI-11	Not Included	0.6812
	mFI-5	Not Included	0.1833
	CCI	0.73 (0.58-0.92)	0.0082

Subgroup analysis of operative patients

Stepwise multivariate logistic regressions, in the same manner as above, were performed among those who received an operative intervention. Among this group, the only independent predictor of mortality was the CCI (OR=4.24; 95%CI: 1.01-17.86; p=0.0491) (Table 7). Similarly, the only independent predictor of discharge home among this group was also the CCI (OR=0.55; 95%CI: 0.33-0.90; p=0.0170). Unlike the cohort overall, the iGCS was not independently associated with either endpoint among those that received an operative intervention.

**Table 7:** Multivariate logistic regressions for primary endpoints among operative cSDH patients CCI: Charlson Comorbidity Index, mFI: modified frailty index, iGCS: initial Glasgow Coma Score, AC/AP: anticoagulation/antiplatelet medication; cSDH: chronic subdural hemorrhage.

	Characteristic	Multivariate OR (95% CI)	P-value
Mortality	Sex	0.93 (0.05-17.53)	0.9615
	Prior SDH	0 (0-infinity)	0.9980
	Craniotomy for SDH	0.34 (0.01-16.75)	0.5840
	AC/AP	infinity (0-infinity)	0.9973
	iGCS	1.17 (0.67-2.02)	0.5831
	SDH Thickness	0.69 (0.41-1.16)	0.1606
	mFI-11	Not Included	0.3296
	mFI-5	Not Included	0.4914
	CCI	4.24 (1.01-17.86)	0.0491
Discharge Home	Sex	2.08 (0.45-9.69)	0.3499
	Prior SDH	0.20 (0.03-1.57)	0.1246
	Craniotomy for SDH	0.58 (0.12-2.81)	0.4943
	AC/AP	0.90 (0.17-4.90)	0.9039
	iGCS	1.02 (0.68-1.54)	0.9128
	SDH Thickness	1.05 (0.94-1.17)	0.3841
	mFI-11	Not Included	0.7254
	mFI-5	Not Included	0.5783
	CCI	0.55 (0.33-0.90)	0.0170

## Discussion

Frailty predicts outcomes in a variety of surgical and non-surgical patients. Despite cSDH being a common neurosurgical condition associated with cerebral atrophy and advanced age, the utility of frailty has not been explored in this population. In this study, we show that frail patients, regardless of scoring system, are less likely to be discharged home compared to their non-frail counterparts. Along with iGCS, the CCI was independently associated with discharge home in multivariate analysis in the cohort overall and was the only independent predictor of this endpoint in patients who received an operation. Similarly, while mortality overall was best predicted by iGCS, the CCI was the only independent predictor of death in subgroup analysis of operative cases. These results indicate that the CCI may have clinical utility in predicting functional outcome in all cSDH patients and mortality among those who receive an operation. 

It is well known that patient comorbidities contribute to poorer prognosis following a neurologic insult. Frailty is an emerging concept that aims to quantify the cumulative effect of these comorbidities in order to more accurately prognosticate outcomes. In neurosurgery specifically, frailty has been associated with poorer outcomes following subarachnoid hemorrhage [5,11], spine surgery [16], intracranial hemorrhage [17], and intra-cranial tumor surgery [18]. Despite this, there are over 215 established frailty indices [7] and no consensus on the optimal scoring system or appropriate cutoffs [19]; something that may be disease and patient-population specific. For example, in our own work we have shown in the same time period and setting as the study herein, the superiority of the mFI-5 and mFI-11 over the CCI in predicting outcomes following angiogram-negative subarachnoid hemorrhage (under review, BJN). This is in contrast to the study herein showing the opposite finding for cSDH. This indicates that despite frailty being associated with poorer outcomes across all of neurosurgery [20], individual scoring systems must be validated for each disease type.

Careful selection of surgical candidates for cSDH evacuation incorporates a variety of factors including hemorrhage size, MLS, age, overall clinical history and exam, and the iGCS. In particular, the iGCS following cSDH is thought to be the factor with the highest prognostic utility [21,22]. In line with this, patients in our study who had an operation were younger, were more likely to have an isolated head injury, a better iGCS, larger cSDH thickness, and lower CCI and mFI-11 scores (Table 3). Likewise, while patients who had an evacuation had longer lengths of stay, they were more likely to be discharged to acute rehabilitation, equally as likely to be discharged home, less likely to be discharged to a nursing home, and trended to have lower mortality compared to their non-operative counterparts. Moreover, among the subgroup of operative patients, the CCI was the only independent predictor of both mortality and discharge location. Together, this reflects careful patient selection for those who are likely to do well after surgery, but given the retrospective nature of this study, we cannot comment on the clinical utility of frailty in patient selection for surgery. Prospective studies with larger samples of those who received a craniotomy or burr hole only may provide more conclusive evidence that the CCI, in addition to better-documented criteria, may be considered for operative candidate selection.

While the mFI-11 is the most commonly used frailty measure in the literature, to date, no study has examined the mFI-11 or mFI-5 among patients with cSDH; an effect that may reflect a publication bias. Furthermore, while there have been previous studies investigating the relationship between the CCI and cSDH prognosis, there are conflicting results with some finding that the CCI is independently associated with cSDH recurrence [23], increased complications [24,25] and long-term mortality [26], while others have shown no such effects [2,27]. Differences in these studies may be attributed to inclusion criteria, with some groups, including our own, examining all cSDH patients, while others only examined certain types of operative patients. For example, Shimizu et al. retrospectively examined 211 patients ≥ 65 years of age who underwent burr hole craniotomy to determine predictors of three month modified Rankin scores, non-home discharge, and unfavorable prognosis. This group showed, in multivariate analysis, that age, nutritional status, and recurrence better predicted endpoints than the clinical frailty score, an established functional measure of frailty, and the CCI. Of note, this group did not include iGCS in multivariate regressions and retrospectively calculated functional frailty scores based on documentation thus limiting the relative clinical utility of their findings [28]. In a similar limitation, we did not include age in multivariate analyses as this variable was included in the CCI score and to do so would incur co-linearity between these variables. Along with this, our goal was to compare frailty with the iGCS and we showed that the iGCS was the most potent predictor of both mortality and discharge home in cSDH overall but was inferior to the CCI among operative patients.

Limitations

This study was chiefly limited by its retrospective design as outlined elsewhere [29] and by a limited sample size. Larger samples may have elucidated more significant effects of frailty on secondary endpoints and perhaps on mortality. In the latter endpoint, there was a trend toward increased mortality with all examined frailty indices. Second, we included all patients who presented with SDH that had a chronic component including mixed, acute-on-chronic, and pure chronic SDH. Larger prospectively obtained cohorts would allow for subgroup analysis of each cSDH type and for stronger comparisons of craniotomy versus burr hole craniotomy.

## Conclusions

While frailty is associated with worse surgical outcomes, the clinical utility of the mFI-5, mFI-11, and CCI versus the iGCS following cSDH is unclear. We show that the iGCS is an overall superior predictor of mortality following cSDH but is outperformed by the CCI after operative intervention. Similarly, the CCI is the superior predictor of discharge home in cSDH patients overall and following an operative intervention. These results indicate that while the iGCS best predicts mortality overall, the CCI may be considered when prognosticating post-operative course and hospital disposition.
